# Food taboo among pregnant Ethiopian women: magnitude, drivers, and association with anemia

**DOI:** 10.1186/s12937-019-0444-4

**Published:** 2019-03-23

**Authors:** Shimels Hussien Mohammed, Hailu Taye, Bagher Larijani, Ahmad Esmaillzadeh

**Affiliations:** 1 Department of Community Nutrition, School of Nutritional Sciences and Dietetics, Tehran University of Medical Sciences-International Campus (TUMS-IC), Tehran, Iran; 2 Unit of Reproductive Health, Addis Ababa City Administration Health Bureau, Addis Ababa, Ethiopia; 3 Endocrinology and Metabolism Research Center, Endocrinology and Metabolism Clinical Sciences Institute, Tehran University of Medical Sciences, Tehran, Iran; 4 Obesity and Eating Habits Research Center, Endocrinology and Metabolism Molecular Cellular Sciences Institute, Tehran University of Medical Sciences, Tehran, Iran; 5 Department of Community Nutrition, School of Nutritional Sciences and Dietetics, Tehran University of Medical Sciences, P.O. Box 14155-6117, Tehran, Iran; 6 Food Security Research Center, Department of Community Nutrition, Isfahan University of Medical Sciences, Isfahan, Iran

**Keywords:** Dietary behavior, Food taboo, Pregnancy, Anemia, Ethiopia

## Abstract

**Background:**

There are pervasive pregnancy-related food taboos and myths (PRFT) in Ethiopia. The evidence, however, is limited on whether PRFT contributes to the burden of maternal anemia. Thus, this study was aimed to determine the magnitude of PRFT, the reasons for adherence to PRFT, and the association of adherence to PRFT with anemia, among pregnant Ethiopian women.

**Methods:**

The study was case-control in design and recruited a sample of 592 pregnant women attending antenatal care in four health facilities in Addis Ababa, Ethiopia. Participants were classified into anemic cases (*n* = 187) and non-anemic controls (*n* = 405) based on their hemoglobin level. PRFT was assessed by the participants’ subjective reporting of avoidance of certain food items during the current pregnancy due to taboo reasons. The specific types of food items avoided and the underlying reasons for the avoidance were also assessed. The relation of PRFT with anemia was evaluated by multiple logistic regression analysis, controlling for covariate factors.

**Result:**

Almost a fifth of the study participants (18.2%) avoided one or more food items due to PRFT. Adherence to PRFT was 26.2 and 14.6% among the anemic and the non-anemic individuals, respectively. The food items most avoided due to adherence to PRFT were green chili pepper, organ meat, and dark green leafy vegetables like spinach, lettuce, kale, and broccoli. The underlying reasons for the adherence to PRFT were largely traditionally held beliefs and misconceptions. After controlling for covariates, PRFT was significantly and independently associated with a higher odds of anemia [adjusted odds ratio (AOR) = 2.12, 95% confidence interval (CI) = 1.32–3.42, *P* = 0.002].

**Conclusion:**

PRFT might be contributing to the burden of maternal anemia in Ethiopia. It is time for public health authorities in Ethiopia to recognize PRFT as a public health risk, strengthen maternal nutrition counseling, and create public awareness of the consequences of PRFT.

**Trial registration:**

ClinicalTrials.gov (NCT03251664), 16 August 2017.

## Introduction

Anemia remains among the major threats to the health and survival of pregnant women. It is often linked to maternal mortality and poor fetal outcomes [1, 2]. The progress of maternal anemia reduction has been slow and less promising globally [1]. Estimates show that 40% of pregnant women were anemic in the year 2016, declining by only three percentage points from the figure in 1990 (43% prevalence) [3]. Reducing the burden of maternal anemia has been a priority public health agenda in Ethiopia, too. However, contrary to what could be expected, the recent years have seen an increasing trend in the prevalence of maternal anemia in Ethiopia, rising from 17% in 2011 to 24% in 2016 [4]. The rise was even more marked among neonates and infants [4].

Anemia is a multi-causal problem, with influences originating from both individual and community levels [2, 5]. The socioeconomic and cultural contexts of the society in which individuals live influence their dietary behavior, including following food taboo [6]. Food taboo forbids eating certain food items; thus, limiting one’s dietary diversity and quality, which may result in poor health and nutritional outcomes. The extent of the practice, as well as the specific food items avoided, varies from one community to another community. However, food taboo is generally more common among rural and less educated communities than among urban and more educated communities [6, 7]. Pregnant women also adhere to food taboos more strictly than non-pregnant women [8, 9]. Pregnancy, in its own, makes pregnant women vulnerable to malnutrition due to the physiological rise in nutrient demand, which may not be adequately met by dietary intake. Thus, further restricting eating due to pregnancy-related food taboos and myths (PRFT) may seriously affect the health of the mother as well as the fetus [7, 8]. Evidence shows higher rates of cesarean section and complicated pregnancy among women adhering to PRFT. Taboo prohibiting the consumption of iron-rich food items, like meat, legumes, or dark green vegetables, might result in anemia in pregnant as well as non-pregnant women [5].

Previous studies done in Ethiopia reported the existence of pervasive PRFT, which may be contributing to the burden of maternal anemia in the country. However, the studies were mainly qualitative [7, 10, 11]; thus, less informative on whether adhering to PRFT was associated with increased risk of anemia, and the extent of this relationship if present. In a recent review of the nutritional habits of immigrant Ethiopian women in Australia, Vasilevski et al. [7] reported that the literature on nutritional knowledge of pregnant Ethiopian women was limited and called for more studies. Thus, in this study, we aimed to determine the magnitude (prevalence) of adherence to PRFT, the reasons for the adherence to PRFT, and the extent of association of adherence to PRFT with anemia status of pregnant Ethiopian women.

## Methods

### Study setting and population

This study was done in Addis Ababa, Ethiopia, among pregnant women attending antenatal care (ANC) in four public health centers and hospitals. Data were collected from October 2015 to February 2016, as part of a project to investigate the dietary and non-dietary determinants of anemia during pregnancy and lactation periods. The project was registered on ClinicalTrials.gov as https://clinicaltrials.gov/ct2/show/NCT03251664.

### Study design, sample size, and sampling procedure

We followed a case-control study design and recruited a total of 592 pregnant women, 187 anemic cases and 405 non-anemic controls. The sample size was determined based on the following assumptions: (i) proportion of exposure among cases 0.27 and controls 0.40, (ii) 1:2 cases to controls ratio, (iii) 10 and 15% non-response rates among cases and controls, respectively, (iv) two-tailed 5% level of significance, and (v) 80% power. Participants were recruited by systematic random sampling scheme based on the ANC client flow of the participating health facilities.

### Study participant selection and allocation

**Fig. 1:**
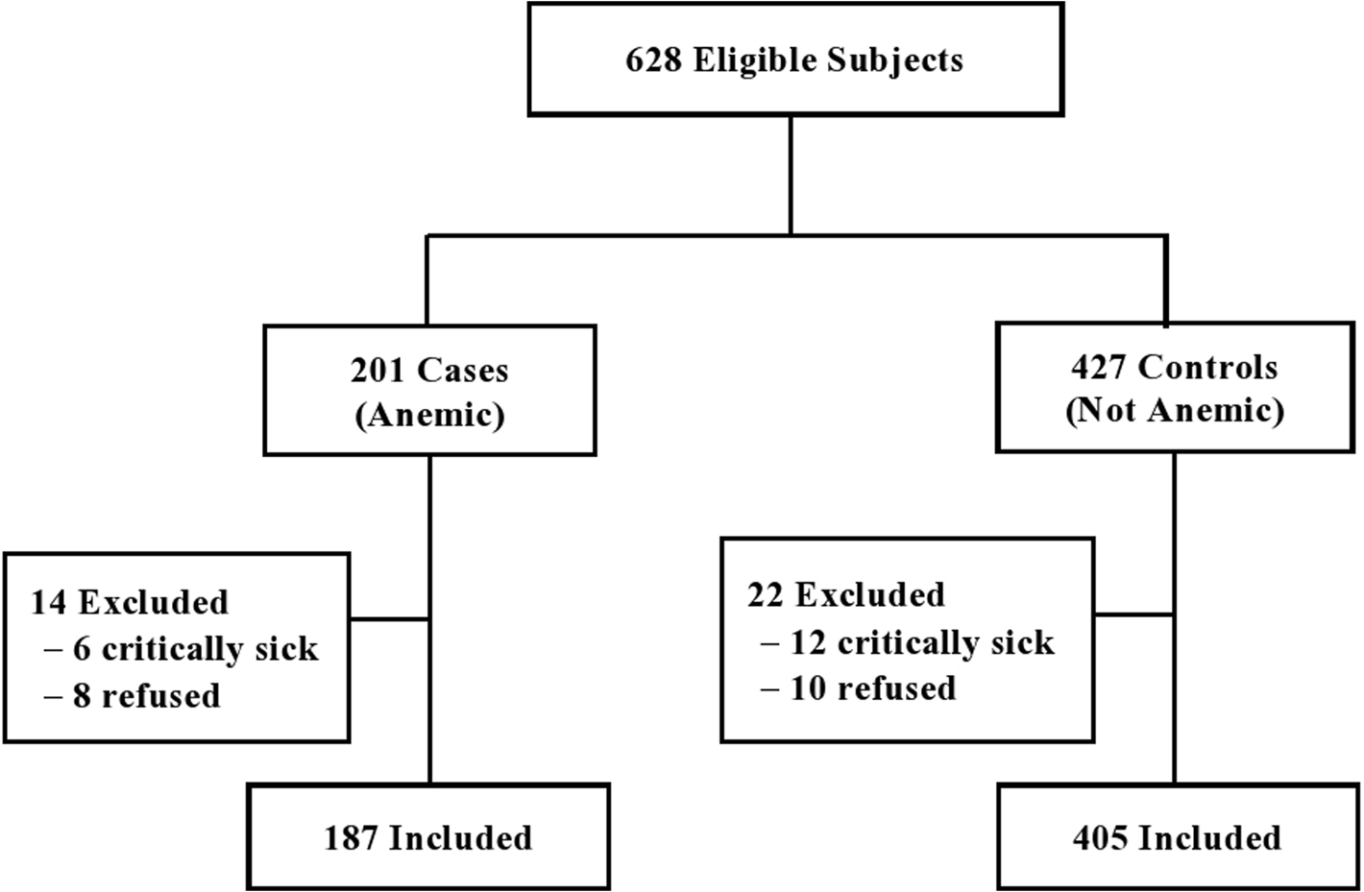
Flowchart of the sample selection procedure

Blood samples from each study participants were taken and examined by the health professionals of the health facilities. Using the blood samples, the participant’ hemoglobin (Hb) level was determined by HemoCue Hb®201 (HemoCue AB, Ängelholm, Sweden). Following the World Health Organisation (WHO) guideline [12], the Hb measures were adjusted for the mean altitude of the study area. The altitude adjusted Hb level was used to allocate participants into cases (anemic, Hb < 11 g/dL) and controls (non-anemic, Hb ≥ 11 g/dL) [12]. The health professionals working in the ANC units of the participating health facilities, together with the study data collectors, evaluated the individual’s eligibility for the study, according to the inclusion and the exclusion criteria of the study. The inclusion criteria were laboratory-confirmed pregnancy status, Hb level measured and anemia status known, and residing in the study area. Individuals were excluded if they had any of the following conditions: (i) severe medical problem requiring hospital admission, (ii) history of major trauma, surgery, blood donation, or abortion in the last 12 months prior to the study, or (iii) currently breastfeeding. Assignment of participants into the two study groups, cases (anemic) and controls (not anemic), was done based on the participants’ anemia status. The cases were recruited sequentially unless they were excluded due to any of the predefined exclusion conditions. For each included case, two consecutive controls were selected from those found not anemic and fulfilled the eligibility criteria. The flow chart of the sample selection and allocation process is presented by Fig. 1.

### Exposure variables

PRFT was the main exposure variable of interest in this study. It was assessed by the participants’ subjective reporting of food items avoided during the current pregnancy due to taboo reasons. To be included in the classification of the PRFT, the food item should have to fulfill all of the following criteria:

(i) The food item should be part of the individual’s diet before becoming pregnant. Thus, food items not consumed by the individual before becoming pregnant were not considered in the PRFT classification.
(ii) The food item should not be potentially unsafe during pregnancy. Thus, potentially unsafe food items like raw meat of any variety, and liver which might also be potential unsafe during pregnancy [13], were not considered in the PRFT classification.
(iii) The food item should not be culturally or religiously prohibited. For example, avoiding pork was not considered as PRFT as it was also a generally prohibited item by the community.

Based on their adherence to PRFT, the study participants were categorized into yes (adhering to PRFT) or no (not adhering to any PRFT). A further assessment of the reasons or beliefs for adhering to and practicing the PRFT was done, using a semi-structured interview guide. A comprehensive assessment of other potential determinants of anemia was also done, including the sociodemographic, reproductive, health, and nutritional status of the participants.

#### Sociodemographic variables

current age, educational status (measured by the highest education completed, and categorized into primary and below, secondary, tertiary and above), religion (categorized into Orthodox Christian, Muslim, others), and income (assessed by the average household monthly income and ranked into three categories: low, middle, high income categories). The classification of the participants into the low, middle and high-income categories was done by the relative position or ranking of the individual compared to the other individuals included in the study.

#### Reproductive health variables

gestational age (the pregnancy duration in months and categorized into three groups: first, second, third trimesters), gravidity (the number of conceptions including history of abortion, if any), and pregnancy interval (the duration from the last delivery to the conception of the current pregnancy).

#### Health/nutrition variables

history of chronic illness (assessed by the presence of any one of tuberculosis, Human Immunodeficiency Virus (HIV), or cancer), intestinal parasites (as reported by the lab stool examination), current use of iron/folate supplements, and hygiene practice (assessed by type of water source and toilet facilities used, and categorized into improved, unimproved). Weight, height, and mid-upper arm circumference (MUAC) measurements were also taken. MUAC is the preferred choice of assessment of the nutritional status of pregnant and lactating women. Thus, based on the MUAC measures, the nutrition status of the participants was classified into wasted (MUAC < 23 cm) and normal (MUAC ≥23 cm) [14].

### Statistical analyses

First, the statistical assumptions for logistic regression analysis were checked. Then, bi-variable analyses between anemia and the exposure variables, including PRFT adherence, were done using Chi-square test of association. The bi-variable analyses were aimed to identify covariates which might influence the main relationship tested, i.e. the relation of PRFT with anemia. Thus, sociodemographic, reproductive, health, and nutrition variables which demonstrated *P* <  0.25 during the bi-variable analyses were included in the final multiple logistic regression analysis. Adjusted odds ratios (AOR), with 95% confidence intervals (CI), were reported based on the results of the multiple regression analysis, done controlling for covariate factors. All analyses were done using STATA version 15 and statistical significance was determined at *P* <  0.05, two-tailed.

## Result

The sociodemographic characteristics of the study participants are shown in Table 1. The majority of the participants (~ 70%) were below 30 years of age, with a mean age of 24.7 years (SD = 2.5 years). Age of the participants ranged from 15 lowest to 39 highest. Religiously, 73.8% of the study participants were Orthodox Christians and 20% Muslims. Only a quarter of the participants completed tertiary and above education levels.

**Table 1:** Sociodemographic characteristics of study participants

Variable	Category	Number (%)
Age group (year)	15–19	20 (3.4)
	20–24	169 (28.5)
	25–29	228 (38.5)
	30–34	90 (15.2)
	35–39	85 (14.4)
Religion	Muslim	119 (20.1)
	Orthodox Christian	437 (73.8)
	Other	36 (6.1)
Marital status	Single	24 (4.1)
	Married	568 (95.9)
Occupation	Employed	114 (19.3)
	Merchant	156 (26.4)
	Housewife	183 (30.9)
	Student	82 (13.9)
	Other	57 (9.6)
Education level	Primary & below	238 (40.1)
	Secondary	198 (33.5)
	Tertiary & above	156 (26.4)

**Fig. 2:**
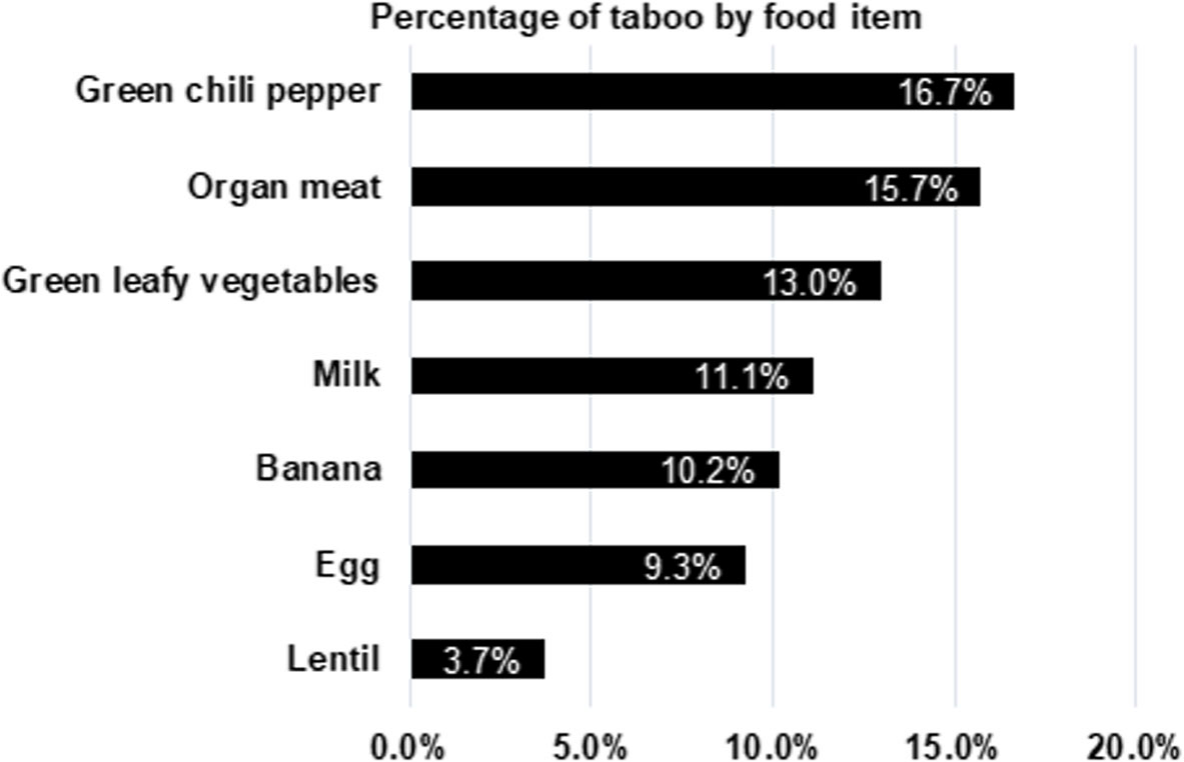
Magnitude (prevalence) of taboo by food items

Overall, 18.2% of the study participants avoided at least one food item during the current pregnancy due to PRFT. The prevalence among the anemic cases and non-anemic controls was 26.2 and 14.6%, respectively. The items most avoided were green chili pepper, organ meat, and dark green leafy vegetables, which were avoided by 16.7 15.7, and 13.0% of the participants, respectively. The list of all food items avoided, with their percentage of avoidance, is found in Fig. 2. The dark green leafy vegetables group included food items like spinach, lettuce, kale, and broccoli. The organ meat group did not include liver, as it is not recommended during pregnancy. The food items least avoided were cereals and grains like wheat, maize, and corn. A third of the participants reported that they used to eat raw meat before becoming pregnant, but not eating currently due to fear of harming the fetus. The reasons for adhering to PRFT were fear of: (i) infection of the fetus as well as the mother, especially when eating vegetables; (ii) large baby and difficult birthing; (iii) offensive vaginal discharge; (iv) abortion; and (v) ‘food sticking’ on the fetus head, especially when eating banana.

Table 2 presents the results of the bivariate analyses between anemia and the exposure variables, including PRFT. Adherence to PRFT was significantly higher in the anemic than in the non-anemic individuals (*P* = 0.001). In terms of educational status, the anemic women were less educated than the non-anemic ones (*P* <  0.001). The proportion of low and middle-income individuals was higher among the anemic women than the non-anemic women (*P* <  0.001). In the second trimester, there were more non-anemic individuals than anemic individuals (38.3% versus 10.7%, *P* <  0.001). In the first trimester, there were more anemic individuals than non-anemic individuals (42.2% versus 23.2%, *P* <  0.001). Wasting (MUAC < 23 cm) was more prevalent in the anemic individuals than in the non-anemic individuals (36.9% versus 23.5%, *P* = 0.001). There was no significant difference between the anemic individuals and the non-anemic individuals by gravidity, pregnancy interval, iron supplement use, intestinal parasites, chronic illness, and hygiene (*P* > 0.05).

**Table 2:** Bivariate analysis of the relation of exposure variables and anemia status

Variable	Category	Cases^a^ N (%)	Controls^b^ N (%)	*P* ^*^
Food taboo	Yes	49 (26.2)	59 (14.6)	0.001
	No	138 (73.8)	346 (85.4)	
Education (year)	Primary & below	105 (56.5)	132 (32.6)	< 0.001
	Secondary	52 (28.0)	146 (36.0)	
	Tertiary & above	29 (15.6)	127 (31.4)	
Income category	Low	73 (39.0)	109 (26.9)	< 0.001
	Middle	70 (37.4)	123 (30.4)	
	High	44 (23.5)	173 (42.7)	
Trimester	First	79 (42.2)	94 (23.2)	< 0.001
	Second	20 (10.7)	155 (38.3)	
	Third	88 (47.1)	156 (38.5)	
Gravidity	<= 2	123 (65.8)	267 (65.9)	0.971
	> 2	64 (34.2)	138 (34.1)	
Pregnancy interval	< 2 years	61 (32.6)	133 (32.8)	0.958
	> 2 years	126 (67.4)	272 (67.2)	
Iron supplement	No	155 (82.9)	317 (78.3)	0.194
	Yes	32 (17.1)	88 (21.7)	
Intestinal parasites	No	174 (93.0)	383 (94.6)	0.466
	Yes	13 (7.0)	22 (5.4)	
Chronic illness	No	176 (94.1)	390 (96.3)	0.162
	Yes	11 (5.9)	15 (3.7)	
MUAC (cm)	<= 23	69 (36.9)	95 (23.5)	0.001
	> 23	118 (63.1)	310 (76.5)	
Hygiene practice	Unimproved	15 (8.0)	36 (8.9)	0.430
	Improved	172 (92.0)	369 (91.1)	

^a^=Cases refer to the anemic group

^b^=Controls refer to the non-anemic group

*P: based on Chi-square test of association

*MUAC*, mid-upper arm circumference

Table 3 presents crude and adjusted ORs of the association of PRFT with anemia. The crude odds of anemia associated with adhering to PRFT was 2.08 times higher compared to that of not adhering to PRFT. To examine whether the association was independent of the influence of the covariates, i.e. confounding effect, AOR was calculated, controlling for the covariate factors identified during the bivariate analyses. There was no significant change in the strength of the PRFT-anemia association after controlling for education level, income, MUAC, gestational age, iron/folate supplement use, and chronic illness. Individuals adhering to PRFT were twice more likely to be anemic than those not adhering to PRFT (AOR = 2.12, 95%CI = 1.32–3.42, *P* = 0.002).

**Table 3:** Crude and adjusted estimates of the association of exposure variables with anemia

Variable		Crude OR		Adjusted OR*	
		OR (95% CI)	*P*	AOR (95% CI)	P**
Food taboo	Yes	2.08 (1.36–3.19)	0.002	2.12 (1.32–3.42)	0.002
	No	Reference		Reference	
Education	Primary	3.48 (2.16–5.62)	< 0.001	3.30 (1.99–5.49)	< 0.001
	Secondary	1.56 (0.93–2.61)	0.089	1.81 (1.05–3.12)	0.034
	Tertiary	Reference		Reference	
Household income^3^	Low	2.63 (1.69–4.11)	< 0.001	2.20 (1.36–3.57)	< 0.001
	Middle	2.24 (1.44–3.48)	< 0.001	1.86 (1.15–3.01)	0.012
	High	Reference		Reference	
MUAC	≤ 23 cm	1.91 (1.31–2.78)	0.001	1.53 (1.01–2.33)	0.047
	> 23 cm	Reference		Reference	
Pregnancy trimester	First	Reference		Reference	
	Second	0.23 (0.13–0.39)	< 0.001	0.25 (0.14–0.44)	0.090
	Third	1.49 (1.00–2.22)	0.049	1.44 (0.95–2.20)	< 0.001
Iron supplement	Yes	Reference		Reference	
	No	0.74 (0.47–1.16)	0.195	1.06 (0.64–1.73)	0.829
Chronic illness	Yes	1.63 (0.73–3.61)	0.233	1.41 (0.60–3.33)	0.430
	No	Reference		Reference	

*AOR*, adjusted odds ratio; *OR*, odds ratio; *CI*, confidence interval; *MUAC*, mid-upper arm circumference

*Multiple logistic regression used to determine statistical significance

**Significant at *P* < 0.05

Some of the covariate variables were also found independently associated with anemia (Table 3). Low education level was associated with a significantly higher odds of anemia, such that individuals with primary and lower education level had a 3.3 times higher likelihood of being anemic, compared to those with tertiary education level (AOR = 3.30, 95%CI = 1.99–5.49, *P* <  0.001). Compared to the high-income individuals, the odds of anemia was 1.86 and 2.2 times higher in the middle and the low-income individuals, respectively. In the wasted individuals, the likelihood of anemia was 1.53 times higher than that of the non-wasted individuals (AOR = 1.53, 95%CI = 1.01–2.33, *P* = 0.047). The odds of anemia was also significantly higher during the third trimester, compared to the first trimester (AOR = 1.44, 95%CI = 0.95–2.20, *P* <  0.001). However, iron supplement use and chronic illness were not significantly linked to anemia (*P* > 0.05).

## Discussion

This study aimed to determine the magnitude adherence to PRFT, the reasons for adhering to PRFT, and the extent at which PRFT was linked to anemia, among pregnant Ethiopian women. We found a considerable portion of the study participants avoiding certain food items of significant importance for the health of the mother as well as the fetus. We also found that PRFT was independently associated with a significantly higher odds of anemia.

Almost a fifth the participants involved in this study avoided one or more food items due to adhering to PRFT. Studies done in Kenya [9] and Mexico [15] among a similar population of women reported PRFT magnitude of 60 and 50.3%, respectively. The low magnitude of PRFT among our study participants might be because it was conducted in an urban setting. Green chili pepper, organ meat, and dark green leafy vegetables were more commonly avoided than other food items like cereals. Previous studies done in Ethiopia [7, 11] as well as other developing countries [8, 9, 15] also reported a high avoidance of meat and vegetables by pregnant and lactating women due to taboo. A third of our study participants reported as they used to eat raw meat but stopped after becoming pregnant. Eating raw meat, particularly strips of fresh beef and lamb in various forms, is a pervasive practice in Ethiopia. However, because eating meat raw is unsafe, we did not consider its avoidance as a taboo or PRFT. Food taboos could hinder individuals from consuming certain food items [7]. This may result in poor dietary quality and diversity, which may subsequently lead to poor health and nutritional outcomes including anemia [7, 8, 16]. Thus, our finding of the association of anemia with PRFT could be in part due to the above reason, i.e. poor dietary quality due to taboo. A further plausible explanation for our finding was that the most avoided food items like organ meat, lentil, and dark green leafy vegetables, are of a better iron profile compared to the least avoided food items like wheat and corn [5].

We believe our finding of the association of PRFT with anemia might, in part, account for the current state of anemia in Ethiopia. Progress in reducing maternal anemia has been less promising in Ethiopia. Instead of decline, it is on the rise across all age groups, regions and sex [4]. Iron deficiency anemia (IDA) was presumed to be the main driver of the global burden of anemia. It was often considered to account for almost half of the global anemia burden [2, 5]. Thus, iron supplementation has taken the centrality of anemia prevention and control interventions in many countries, including Ethiopia [17]. However, recent evidence has shown that the contribution of IDA to anemia is not as high as the widely held presumption [18, 19]. It could be even insignificant in Ethiopia due to a set of dietary and genetic conditions, unique to Ethiopians [20–22]. However, notwithstanding the importance of iron supplementation during pregnancy, our finding of an independent association of PRFT with anemia might imply that PRFT needs to be addressed to curb the burden of anemia in Ethiopia.

Though not the main objective of the study, use of iron supplement was not found significantly associated with anemia. As iron is an important nutrient for erythropoiesis [5], use of iron supplement is normally expected to correlate with an improved hemoglobin level. Notwithstanding that, there are many plausible explanations for the finding. First, our analysis did not take into account the adherence and duration of the iron supplement use. These factors will influence the level of hematologic response to iron supplement use [5]. Second, most of the participants may not have been iron deficient. Some studies have shown that iron deficiency is not a major problem in Ethiopia [19, 21, 23]. Third, it could be related to some unique environmental, genetic, and dietary factors which might influence the absorption, transport, and metabolism of iron [20, 22, 23]. The validity of the WHO hemoglobin cutoff points for highland populations, including Ethiopia, is also questionable [24, 25]. Thus, as the case of anemia in Ethiopia has not been well investigated, it would be hard to state the specific reason that is most responsible for the lack of association of iron supplement use with anemia among our study participants.

This study has important policy implications. There is low awareness among Ethiopian women about the pregnancy-related changes in nutrient demand and the dietary recommendations to meet the changing demands [7]. This might be due to the poor integration of nutrition services with the existing health care system in the country. For example, nutritionist or dieticians are not included in the Ethiopian hospitals’ workforce [26]. The dietary information provided by other professionals might be incomplete and vague. This might also be the reason for the low awareness of Ethiopian mothers on the negative consequences of food taboo [7, 11]. Thus, it stands important for health facilities in Ethiopia to consider integrating nutrition care and support with the existing system. ANC visits, in particular, are great opportunities to reach pregnant women, address PRFT, and avert its consequences on the mother as well as the fetus. Pregnancy is a critical period for both the mother and the fetus as the effects of nutritional deficiencies during this period could be irreversible [17]. Compared to non-pregnant women, pregnant women are more receptive to nutritional advice and also more willing to change their eating behavior [7, 16]. In addition to strengthening the institutional nutritional services, public awareness creation also stands an important consideration. In general, Ethiopian women are already at higher risks of complicated pregnancy [7], maternal mortality, and anemia [4]. PRFT may further aggravate the vulnerability to these risks. Thus, it is time for the Ministry of Health of Ethiopia to consider food taboo as a public health challenge and design appropriate interventions.

The study has some limitations worth presenting to the reader. The design, case-control, precludes making causal inference or ruling out reverse causality. It would be beneficial to repeat these analyses using data from prospective studies to strengthen our conclusions. We did not evaluate the association of anemia with each of the specific food items avoided. Thus, the strength of the PRFT-anemia association may change by the specific food items avoided as hematologic response to food varies by the type of food item. Thus, the estimate we reported might not be applicable to any of the specific food items. The study was done using hospital-based samples, which would be limiting its generalizability to the whole population. The women attending ANC visits might be different from those not attending ANC, which would further undermine the generalizability of the findings. We did not collect data on the pattern of food replacement due to the PRFT. Thus, we could not examine whether the replacement, if any, affects the association of PRFT with anemia. Future researchers are encouraged to investigate the pattern of replacement as well as its influence on health in general and anemia in particular. The main strength of the study was that it addressed a neglected problem of public health importance. The comprehensive adjustment for various factors might have minimized the possibility that the observed association was confounded.

## Conclusion

There was a concerning level of adherence to PRFT among pregnant Ethiopian women living in Addis Ababa. Green chili pepper, organ meat, and dark green leafy vegetables were the most avoided food items, with the reasons for the avoidance being largely traditionally held myths and misinformation. PRFT was significantly and independently associated with a moderately higher likelihood of anemia. Thus, PRFT might be among the factors contributing to the burden of maternal anemia in Ethiopia. Further prospective studies are warranted to provide better evidence on the exact contribution PRFT to the burden of anemia. However, as adherence to PRFT is often harmful, it is time for public health authorities in Ethiopia to recognize and address the problem. To that end, strengthening maternal nutrition counseling and creating public awareness of the consequences of PRFT represents worthy of consideration.

## Abbreviations

ANC: Antenatal care
AOR: Adjusted odds ratio
CI: Confidence interval
Hb: Hemoglobin
MUAC: Mid-upper arm circumference
OR: Odds ratio
PRFT: Pregnancy-related food taboo
SD: Standard deviation
WHO: World Health Organization
